# Shaping in the Third Direction: Self-Assembly of Convex Colloidal Photonic Crystals on an Optical Fiber Tip by Hanging Drop Method

**DOI:** 10.3390/polym16010033

**Published:** 2023-12-21

**Authors:** Ion Sandu, Iulia Antohe, Claudiu Teodor Fleaca, Florian Dumitrache, Iuliana Urzica, Simona Brajnicov, Romulus Iagaru, Bogdan Alexandru Sava, Marius Dumitru

**Affiliations:** 1 National Institute for Lasers, Plasma and Radiation Physics, Lasers Department, 409 Atomistilor Street, 077125 Magurele, Romania; ion.sandu@inflpr.ro (I.S.); iulia.antohe@inflpr.ro (I.A.); claudiu.fleaca@inflpr.ro (C.T.F.); florian.dumitrache@inflpr.ro (F.D.); iuliana.iordache@inflpr.ro (I.U.); simona.brajnicov@inflpr.ro (S.B.); 2 Romanian Academy of Scientists (AOSR), 54 Splaiul Independenţei, 050094 Bucharest, Romania; 3 Lucian Blaga University of Sibiu, 10 Victoriei Bvd., 550024 Sibiu, Romania; iagaru@uamsibiu.ro; 4 University “Politehnica” of Bucharest, 313 Splaiul Independentei, 060042 Bucharest, Romania

**Keywords:** self-assembly, blazing, convex, colloidal, photonic, crystal, fiber

## Abstract

High-quality convex colloidal photonic crystals can be grown on the tip of an optical fiber by self-assembly using the hanging drop method. They are convex-shaped, produce the diffraction of reflecting light with high efficiency (blazing colors), and have a high curvature. The convex colloidal crystals are easily detachable and, as free-standing objects, they are mechanically robust, allowing their manipulation and use as convex reflective diffraction devices in imaging spectrometers. Currently, the same characteristics are obtained by using gratings-based structures. The optical fiber/colloidal crystal interface is disordered; thus, no light diffraction can be registered. The ordering at this interface was highly increased by forming a polystyrene spacer on the optical fiber tip, which served as a self-assembly substrate for silica colloid, as a mechanical bond between the fiber and the crystal, and as a filler reservoir for an inverse-opal synthesis. The silica opal-like grown on the optical fiber tip can be transformed into a high-quality polystyrene (blazing colors) inverse-opal by using the polystyrene spacer as a filler. We found that the colloidal crystal axisymmetric self-assembles onto the optical fiber tip only if a maximum volume of the colloid drop is settled on a flat end of the polystyrene spacer.

## 1. Introduction

The technological concept of “lab-on-chip” [1], which is capable of extraordinary performances, such as the use of small volumes of analyte, multiple laboratory functions, compactness and increased portability, and low exploitation cost compared to current technologies, has been combined with the unique advantages of optical fibers (OFs), such as their intrinsic ability to conduct light at a distance, microscopic coupling section, high aspect ratio, and their biocompatibility [2]. This has led to the emergence of an exciting new field of research and technological applications called “lab-on-fiber” (LOF) [3,4,5]. The aim of this LOF technology is to develop a new class of sensors with advanced miniaturization and overall performance characteristics for applications in chemical and biological sensing. The key idea in LOF technology is the use of an optical fiber as a platform for the micro and nanoscale fabrication of integrated functional components (labs), with the control and collection of laboratory-generated information performed through the optical fiber. The laboratories in LOF sensors can be nanoplasmonic devices, metasurfaces, or photonic crystals (PCs) that interact with light at the sub-micron or nano level, producing effects such as surface-enhanced Raman scattering (SERS), surface plasmon resonance (SPR) or Bragg diffraction. Depending on the specific location where functional materials at the micro and nanoscale are integrated, “lab-on-fiber” technology is classified into three main paradigms: lab around fiber, lab in fiber, and lab on tip (where functional materials are integrated onto the optical fiber tip) [6]. A significant body of research has been required to identify fabrication strategies capable of working properly on unconventional substrates involving curved, small-cross-section, and high-aspect-ratio surfaces, such as those of optical fibers, which are quite different from the flat surfaces currently used by high-performance nanotechnologies. Certainly, the flat tip of an optical fiber is an attractive platform for sensor applications because it can be a kind of “plug-and-play” low-invasiveness media sensor. Controlling light at the nanoscale of these sensors requires control of the geometrical characteristics of the structures through which light travels. This can be undertaken either using the high-performance but expensive top-down [6,7] class of technologies, or the cheap but much less precise bottom-up [6,7,8] class of techniques and technologies. The first class includes electron-beam lithography (EBL) and focused ion beam (FIB) milling, and the second includes chemical vapor deposition (CVD), sol-gel processing, bio-assisted synthesis, electroplating, flame-spraying synthesis, atomic or molecular condensation, and self-assembly (SA). Each class may also be divided into two subclasses. The first deals with the preliminary fabrication of nanostructures on planar substrates via standard processes, and their subsequent transfer onto the fiber tip. The second subclass includes the direct writing in which nano-structuring processes (deposition and patterning) are directly realized on the optical fiber end-facet. Each subclass has its own performances and disadvantages. The first subclass takes advantage of standard nano-fabrication processes. Their main limitation lies in the final transferring step (structure detaching, bonding, welding), which determines both the fabrication yield and the performance of the device. The second involves strict requirements for controlling the fiber positioning and handling within fabrication equipment, which is conceived for operations with standard planar substrates.

In this article, we propose a synthesis method involving the placement of a colloidal photonic crystal directly onto the tip of an optical fiber through the self-assembly (SA) phenomenon [9]. The SA method represents an easier, faster, and cheaper alternative to nano-lithography for realizing micro- and nanostructures. Furthermore, SA approaches allow the creation of ordered patterns on large areas and, in particular, on several optical fibers in parallel, paving the way for the mass production of fiber-tip devices. However, for obtaining full control of the characteristics of the structure in order to integrate it on the fiber tip, the autonomous organization of micro and nanoparticles (NPs) in ordered ensembles is crucial. Unfortunately, “most of the SA processes fail when they are required to operate on very confined areas such as the optical fiber tip,” as P. Vaiano et al. mentioned in a review article [7].

However, there are few methods able to self-assemble a colloidal crystal onto the optical fiber tip. To cite examples, F.L. Yap et al. (2012), I. Antohe et al. (2017) used drop coating [10,11], H. Yan et al. (2009) used directed liquid coating [12], and P. di Palma et al. (2017, 2019) used dip coating [13,14] to fabricate this kind of colloidal photonic crystals, and showed that these crystals are sufficiently ordered to generate Bragg light diffraction. In a previous article [15] we showed that a high-quality colloidal photonic crystal can be grown from a hanging colloidal drop on a semi-infinite flat substrate (the substrate is much more extended than the drop size). This article presents the self-assembly of (sub)micron spheres from a colloidal drop hanging on an optical fiber tip (the substrate is less extended than the drop size) as opal-like colloidal crystals that show a larger curvature and a much smaller lateral size, of hundreds of micrometers versus the 5–7 mm in those prepared on a semi-infinite substrate. This could allow them to be easily integrated in hypodermic needles and catheters for in-vivo theranostic and point-of-care diagnostics. In addition, they could work as colloidal crystal tunable lasers coupled with an optical fiber. These opals can also be transformed into functional inverse opal photonic crystals.

## 2. Materials and Methods

### 2.1. Materials

We purchased SiO_2_ sub-micron spheres with a mean diameter of 0.264 µm, 5% *w*/*v* and polystyrene spheres measuring 20.00 μm, 10% *w*/*v* as aqueous colloidal solutions from microParticles GmbH, Berlin, Germany, and either used them as they were or diluted with deionized water. Other micron and sub-micron polystyrene and silica spheres were also used.

Polystyrene flakes from Sigma-Aldrich Chemie GmbH, St. Louis, MO, USA, Product Number: 331651, melting temperature 240 °C, were dissolved in acetone, 20% *w*/*v*, or melted to 270 °C.

Optical probes were manufactured by cutting TEQS multimode optical fibers (Thorlabs, Dortmund, Germany) with a core diameter of 400 µm into pieces measuring 10 cm. In some of the probes, a zone of 1 mm was constructed at one side by mechanically removing the optical fiber jacket. In other cases, even the uncovered polymer cladding was dissolved in acetone, followed by drying the optical fibers with dust-free tissues.

Standard and commercially available micro-syringes, 1 mL in volume (needle incorporated), needle G 29 (377 µm outer diameter, 184 µm inner diameter, 76 µm wall thickness), were used for depositing colloidal solution onto the optical fiber tip.

### 2.2. Methods

#### 2.2.1. The Synthesis of Polystyrene Spacer on the Tip of an Optical Fiber

##### The Synthesis of Polystyrene Spacer on the Tip of an Optical Fiber from Polystyrene/Acetone Solution

The optical fiber probe was placed with the tip down in a vertical position. A droplet of polystyrene/acetone 20% *w*/*v* solution was formed on syringe tip, and it was gently transferred by touching the optical fiber tip. The probe was heated for 1 min at T = 270 °C. This maneuver was repeated a few times until an axisymmetric polystyrene solid droplet was formed at the end of the optical fiber.

##### The Synthesis of Polystyrene Spacer on the Tip of an Optical Fiber from Melt Polystyrene

A small volume of polystyrene powder was deposited onto a slide glass and heated to melt to 270 °C. It formed a 1–2 mm thick liquid melt film. Kept in a vertical position, the optical fiber probe’s tip was sunk for a few seconds in the polystyrene melt film and the optical fiber probe was retracted to the outside environment and kept in a vertical position. A small spheroid formed on the optical fiber tip. If the optical fiber probe tip is quickly pressed onto a cold glass slide after the fiber is extracted from the melt, the polystyrene spacer is flattened.

#### 2.2.2. Opal-like Colloidal Photonic Crystal Self-Assembled on the Tip of an Optical Fiber

The optical fiber probe (simple tip or with polystyrene spacer attached) was placed with the tip down in a vertical position. A droplet of colloidal solution was formed on syringe tip and was gently transferred by touching the optical fiber tip or polystyrene spacer. The volume of colloidal drop deposited on the optical fiber tip should be near its maximum. This maximum depends on the nature and fiber diameter. For the raw core of 400 μm glass fiber, the maximum volume of colloidal drop that can be hung is 3 μL. The gentle manipulation of the syringe piston allows the formation on the needle syringe’s tip of droplets with volumes varying between 0.1 μL and around 5 μL. The shape of colloidal crystal and quality of sphere self-assembly do not depend on the volume of the hangedcolloid drop. However, the colloidal crystal thickness proportionally varies with the volume of hanging colloid drop. The drop was left to dry in the normal conditions of the laboratory, with the process usually taking place within 30–60 min.

#### 2.2.3. Inverse Opal Colloidal Photonic Crystal Self-Assembled on the Tip of an Optical Fiber

A droplet of SiO_2_ colloidal solution was deposited onto the polystyrene spacer as in the Section 2.2.2, finally resulting in a SiO_2_ opal-like adhered on the polystyrene solid droplet. In the same vertical position, the probe was heated in a furnace at T = 270 °C for 15 min. Finally, the optical fiber tip which has deposited the colloidal crystal was immersed in 25% *w/v* HF aqueous solution for 15 min, rinsed with distilled water, and allowed to dry.

### 2.3. Investigations

Macro-scale observations of the as-synthesized patterned colloidal crystals were performed using a zoom camera and optical microscopy. A scanning-electron microscope (SEM) (Apreo S Thermo Fisher Scientific, Auburn, AL, USA) and an atomic force microscope (AFM) XE-100 (Park Systems Inc., Santa Clara, CA, USA) were used to observe the structures and morphologies of the self-assembled patterned colloidal crystals. The UV–Vis transmittance and reflectance spectra were acquired by using optical fibers that were connected to an AvaLight-DHc light source (spot size ~200 µm) and an AvaSpec-ULS2048CL-EVO high-resolution spectrometer, both from Avantes, Apeldoorn, The Netherlands.

## 3. Results

**Figure 1:**
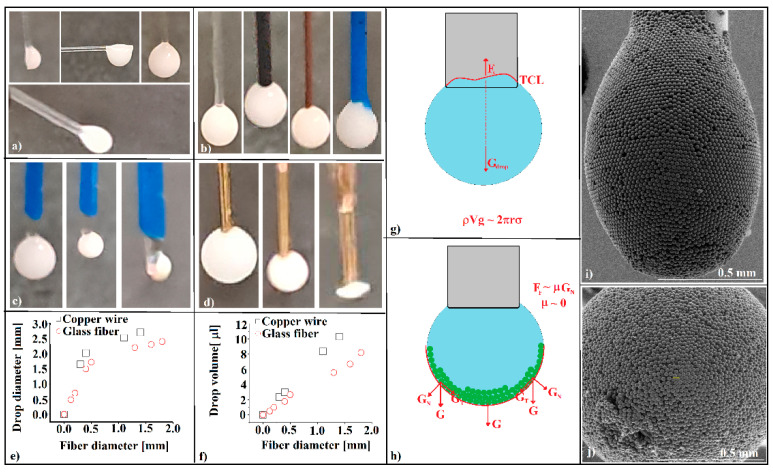
(**a**) Colloidal solution droplets resting in the end zone of an optical fiber (400 μm core); (**b**) colloidal solution droplets hanging on diverse fibers: glass, carbon, copper, polymer; (**c**) colloidal droplet hanging on the optical fiber tip forming a non-axisymmetric colloidal photonic crystal through liquid evaporation; (**d**) colloidal droplet hanging on the metallized (Au) optical fiber tip, forming an axisymmetric colloidal photonic crystal; (**e**) the relation between the diameter of the maximum volume of a colloidal drop and fiber diameter for a glass fiber and a copper wire; (**f**) the relation between the drop maximum volume of a colloidal drop and fiber diameter for a glass fiber and a copper wire; (**g**) schematic configuration of forces at equilibrium in a hanging droplet; (**h**) schematic configuration of colloidal spheres self-assembly on a fiber tip; (**i**,**j**) SEM side and front images, respectively, of a polystyrene sphere (20 μm) opal-like colloidal crystal self-assembled on an optical fiber tip.

A free water drop is spherical as long as it is completely isolated from any external influence. When the drop rests on a solid surface, it takes a near-spherical or a cape-of-sphere shape, depending on the adhesion force intensity between the water molecules and the substrate’s surface atoms, if gravity action can be neglected. If not, the drop flattened to a “pancake,” like onto a horizontal substrate, takes a tear-like shape onto an inclined shape, or a multitude of shapes if the drop does not rest but hangs on the substrate [16,17,18,19,20]. These shapes are usually only found for flat and semi-infinite substrates. The “Gibbs edge effect” (Gibbs inequality) [21] also changes the shape and equilibrium conditions of a drop that rests or hangs on the substrate. It is very important to manage the shape of the drop because if it is not a simple liquid and contains many small, identical objects, then physical phenomena such as agglomeration [22], flocculation, crystallization [23], coalescence, or self-assembly (which can be seen as a spectacular and complex form of agglomeration) are strongly influenced by the drop shape and, especially, by the relative position between the solid particles in the drop and both the substrate and the air/liquid interface, as well as by particle movement relative to the gravity direction and sense [15]. A droplet of sub-micron-sphere colloid, which can contain billions of such identical objects, can be deposited on the head of a glass fiber in different positions (Figure 1a), by forming a droplet at the needle end of a microsyringe and transferring the droplet onto the tip of the glass fiber by a simple touch. However, the specific contribution of our research is that during self-assembly, the colloidal drop is placed at the fiber tip and is axisymmetric in relation to the fiber length and gravity direction. In a similar way, the droplet can be axisymmetrically deposited on glass or other kinds of fiber, such as carbon, copper, or polymeric fiber (Figure 1b), or the volume of the pre-deposited drop can be increased (up to a point) by touching it to a new drop forming on the tip of the microsyringe (Figure 1b).

It appears that that if we want to obtain an axisymmetric colloidal crystal on an optical fiber tip, then an axisymmetric colloidal drop must be deposited initially. Surprisingly, it rarely happens (Figure 1c). We found that if we start from a drop with an almost maximum volume, deposited on an optical fiber that has a pre-deposited gold film (Figure 1d), or if the droplet is deposited on the head of the transversely cut optical fiber (in contact with the fiber jacket), then the resulting self-assembled colloidal crystal is axisymmetric in relation to the optical fiber. A lower wettability of the fiber seems to facilitate the axisymmetric positioning of the crystal, whereas a higher wettability moves the crystal from the axis of symmetry. This behavior occurs in the final stage of drying and is due to non-axisymmetric capillary forces whose size depends on substrate wettability. We found that the maximum volume of the colloidal drop that can be attached to the optical fiber roughly checks, in an expected way, the necessity of equality between the drop weight and the capillary force exerted between drop and fiber (Equation (1)), (Figure 1e,f).

*ρVg* ≈ *2πrσ* (1)

where *ρ* is the drop density, *V* is its volume, *g* is the gravity constant, *r* is the fiber radius, and *σ* is the surface tension of the liquid forming the drop.

These important forces involved in the mechanical equilibrium of the colloidal droplet hanging from the tip of the optical fiber, namely gravity and capillary force interplay at the boundary between the liquid, the air, and the optical fiber (triple contact line, TCL) [24] (Figure 1g). Deviation from the ideal shape of the triple contact line (a circle) caused by surface defects in the optical fiber (roughness and chemical impurities adsorbed on the surface) leads to deviation from the axi-symmetry of the self-assembled colloidal crystal. However, this sinuous triple-contact-line boundary has its own (local) dynamics during the solvent’s evaporation from the droplet, which leads to the change over time of the capillary force’s direction, inducing their deviation from collinearity with gravity. In the freshly deposed drop, the sub-micron spheres are in a highly disordered state. As the solvent evaporates, those spheres agglomerate and then depose under the gravity’s action on the bottom zone of the liquid–air interface. The number of layers continuously increases up to the end of the evaporation. Regarding the self-assembly phenomenon, it benefits the “hanging drop” configuration [15] in two important respects. The first is the tangential component of the weights of the colloidal spheres (Figure 1h). Once the spheres settle on the liquid/air interface or are in direct contact with others that are already deposited [15], this component confines the spheres in a compact colloidal (opal-like) crystal of good quality. The second factor, the zero static frictional force between the spheres and the liquid/air interface [15,25,26], leads to the possibility of the whole system being permanently reconfigured (until “freezing”), making it equivalent to a defect-free ideal substrate. The self-assembly quality through the “hanging drop” method is illustrated in Figure 1i,j, where colloidal polystyrene spheres (20 μm in diameter) form a round (spheroid) opal-like crystal on the tip of a 400-micrometer-core optical fiber. The sphere, spheroid, and hemisphere macroscopic shapes that the colloidal crystal formed by this method can take depend both on the diameter and the nature of the spheres in the colloidal solution and on the initial concentration of the colloidal solution. We also found that while small (nanometer and sub-micron) colloidal spheres tend to form compact crystals, the large spheres (microns or tens of microns in diameter) concentrate into a thick shell.

**Figure 2:**
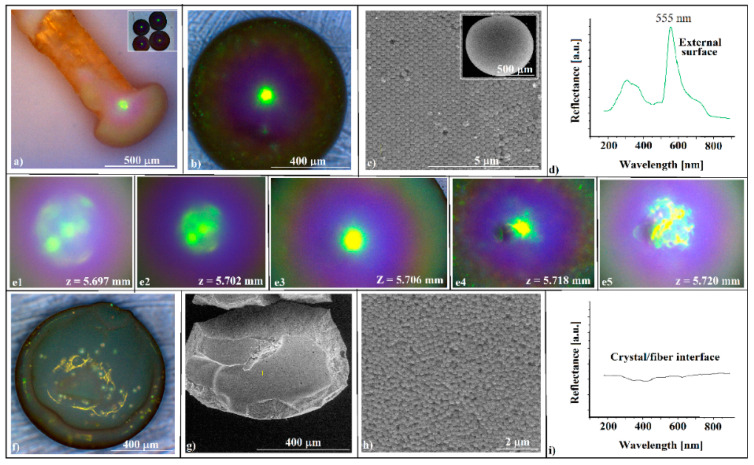
(**a**) Optical microscopy side-view image of a SiO_2_ colloidal crystal self-assembled on an optical fiber tip, inset—a group of free-standing SiO_2_ colloidal crystals; (**b**) optical microscopy front-view image of a SiO_2_ colloidal crystal (convex surface); (**c**) SEM images, with different magnifications, of outside (convex) surface of the SiO_2_ opal-like colloidal crystal; (**d**) UV-Vis retro-reflection spectrum performed on the SiO_2_ colloidal crystal convex surface; (**e1**–**e5**) optical microscopy front-view images of a SiO_2_ colloidal crystal during crystal—objective lens distance variation; (**f**) optical microscopy image of a SiO_2_ colloidal crystal (optical fiber interface); (**g**,**h**) SEM images of the SiO_2_ opal-like colloidal crystal (optical fiber interface); (**i**) UV-Vis retro-reflection spectrum of the optical fiber interface—detached opal.

The SiO_2_ (264 nm in diameter) colloid forms through self-assembly on the fiber tip opal-like colloidal photonic crystals, which are easily detachable and, as free-standing objects, they are mechanically robust, allowing their manipulation and use as components in some optical experimental set-ups. This is due to the high level of thickness of the colloidal crystals; they do not show cracks or voids in their volume or on their surface (Figure 2c,g). They are convex-shaped, and they produce the diffraction of reflecting light with high efficiency (blazing colors can be seen on optical microscopy (Figure 2a,b,e), and have a high curvature (sub-millimeter sizes)). These characteristics could make them useful as convex reflective diffraction devices in imaging spectrometers; currently, the same characteristics are obtained by using gratings-based components in the Offner and Offner–Chrisp optical design geometries [27].

The SEM investigations (Figure 2c) show a hexagonal, close-packed (hcp) array of silica spheres forming large and well-ordered domains, and the UV-Vis reflectance spectrometry reveals the existence of a sharp peak around 555 nm, which corresponds to (111) family planes (Figure 2d), according to the Bragg equation of light diffraction (Equations (2) and (3)).

(2) $$m\lambda =2d_{hkl}\sqrt{n_{eff.\;}^{2}-{sin}^{2}\theta \;}$$

where *λ* is the wavelength of the reflectance peak maximum (i.e., the position of the photonic band gap), *hkl* is the interplanar spacing between the *hkl* planes, *m* is the order of the Bragg diffraction, *n_eff_*. is the average refractive index of the photonic structure, *θ* is the angle between the incident light and the surface normal, and *n_eff_*. is given by (Equation (3)),

(3) $$n_{eff.}={fn}_{{SiO}_{2}}+\left(1-f\right)n_{air}$$

where *f* is the filling factor, and *n_SiO_*2*__* and *n_air_* are the refractive indices of the SiO_2_ and the air, respectively.

The theoretical band-gap was found by using the following values in Equations (2) and (3): 1.42 for silica, 1.00 for air refractive indices, f = 0.74 as filling factor, m = 1, θ = 0. It was placed at λ 561 nm, which is close to our measured value.

We would like to point out an interesting phenomenon that was observed with optical microscopy, namely the change in color (green to yellow) of the central area of the convex crystal (Figure 2e) as the distance z between the crystal and the microscope objective increases, revealing a possible correlation between the wavelengths of diffracted light and its corresponding focal positions in a convex diffractive reflection mirror.

**Figure 3:**
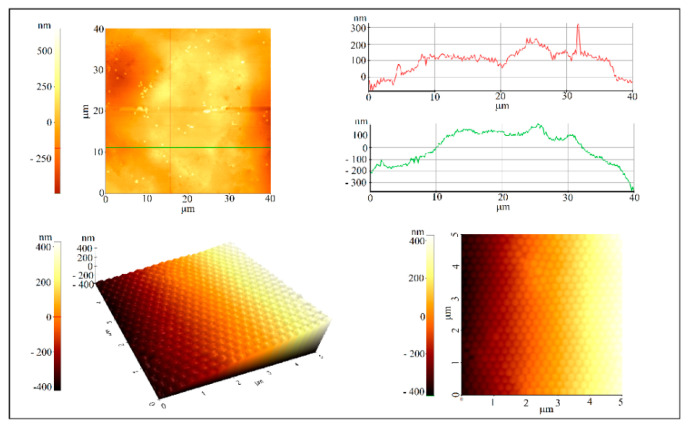
AFM images of or near the SiO_2_ colloidal crystal apex.

No intense light diffraction/reflection was observed on the surface in contact with the optical fiber tip (Figure 2f). This surface is less ordered (Figure 2g,h) and, consequently, no reflection band for the inside surface was detected (Figure 2i). Attempts to increase the ordering of the spheres at this interface by classical approaches, such as slow evaporation or mixing the colloidal solution with different percentages of ethanol, failed. Each time, the outer surface remained the same or its ordering increased slightly, but the interface ordering did not improve. The self-assembly of a colloidal solution sphere on the tip of a fiber produces two surfaces with a completely different ordering quality. The quality of the external surface is high, as shown on the AFM images (Figure 3), and the order of the colloidal crystal/fiber interface was almost non-existent.

**Figure 4:**
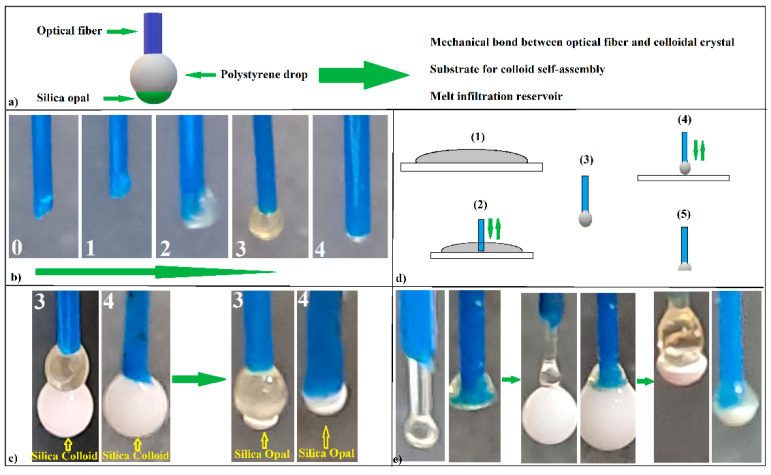
(**a**) Schematic of PS spacer roles; (**b**) images of PS spacer formation from PS/acetone solution on an optical fiber tip; (**c**) colloidal crystal self-assembly onto PS spacers formed from solution; (**d**) schematic of PS spacer formation from melt PS; (**e**) colloidal crystal self-assembly onto PS spacers formed from melt PS.

With a disordered optical fiber/colloidal crystal interface, the hanging drop method seems unsuitable for the self-assembly of a colloidal crystal directly on the optical fiber head to be used as an opto-chemical sensor (the shifting of the (111) reflection band with the variation of the refractive index of the liquid in which the optical fiber head is inserted). However, the transformation of SiO_2_ opal into an inverse opal that retains its outer surface quality can extend the range of objects that can be used as convex diffraction devices. Because our attempts to obtain an inverse opal by solution infiltration failed, resulting in a poor-quality, collapsed or skin-covered inverse opal, with an unsatisfactory adherence, the “inverse infiltration” [28] of a melt seemed to be a better solution. A polystyrene spacer between the fiber tip and colloidal crystal might solve the poor mechanical resistance and adherence to the optical fiber and, at the same time, it can serve as a self-assembly substrate for silica colloid and as a filler reservoir for an inverse-opal synthesis (Figure 4a).

It is complicated to place exactly the right amount of polystyrene filler on the optical fiber head (a few micro-grams) starting from small solid pieces. An acetone–PS solution allows the exact dosing of the filler onto the fiber tip. However, the acetone solvent must be totally removed by boiling because the alternative free evaporation of the solvent is an extremely slow process. Moreover, this process must be performed before the colloidal crystal formation in order to avoid their deviation from axis symmetry, which is induced by the boiling turbulences. Thus, eventually, a small solid polystyrene sphere, axisymmetric with the optical fiber, should be obtained. To this end, we started with a 20 wt.% solution of PS dissolved in an acetone drop that was attached to the end of the OF. Unfortunately, in this case, the same competition between the phenomenon of wettability and the action of gravity occurred, as shown in Figure 1c and, consequently, the final PS drop was not aligned with the axis of the optical fiber. However, by successively applying several acetone-dissolved PS drops (Figure 4a), treating the system at T = 270 °C for 1 min after each applied drop, we obtained, after the third or fourth drop, a solid PS sphere that was axisymmetrically attached to the end of the optical fiber (drop 3 (Figure 4b)). Of course, for more diluted PS-acetone solutions, the number of required drops number will increase. Any supplementary PS–acetone drop will induce the near-complete detachment of the PS melt, leaving behind only a small polystyrene hemisphere attached to the optical fiber (drop 4 (Figure 4b)). Now, both droplet 3 and droplet 4 can be used as substrates for the self-assembly of SiO_2_ colloidal spheres by the hanging drop method (Figure 4c). A second approach (which is much easier) is to melt (T = 270 °C) some polystyrene flakes deposited onto a glass slide (Figure 4d step one) and to insert and remove (for a few seconds) the end of the optical fiber in a vertical position in the melted polystyrene film (Figure 4d, step two). At the end of the optical fiber, a polystyrene sphere forms with a diameter that is slightly larger than that of the optical fiber (Figure 4d, step three). If the process is continued by quickly pressing the melted polystyrene sphere before it casts onto a cold glass slide (Figure 4d step four), a polystyrene spacer with a flat surface parallel with the optical glass fiber’s end face is obtained (Figure 4d step five). Both the spherical and the flat (end surface) parts of the spacers can be used as substrates for the silica colloid self-assembly (Figure 4e).

Surprisingly, this time, the UV-Vis measurements recorded a (111) diffraction band on both the outside surface, at λ = 555 nm, and the optical fiber interface of the silica colloidal crystals self-assembled onto the polystyrene spacer, at λ = 550 nm, respectively. These crystals were investigated after their easy detachment from the PS spacer.

**Figure 5:**
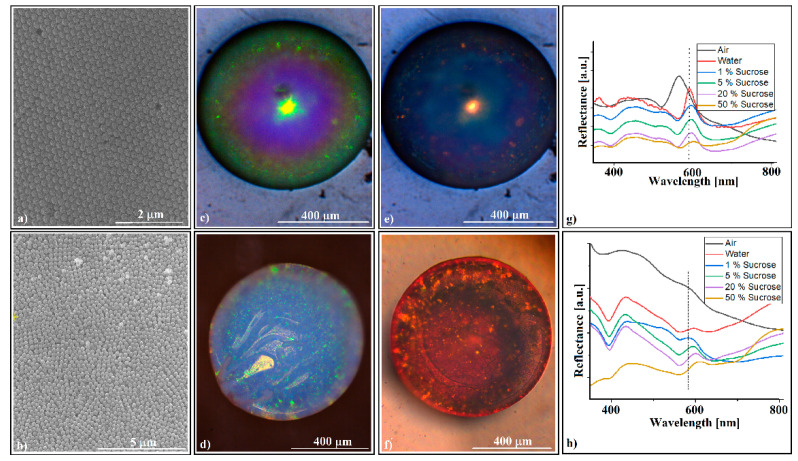
(**a**,**b**) SEM images of SiO_2_ opal-like colloidal crystal surfaces self-assembled on the PS spacer and subsequently detached from the optical fiber tip: (**a**) convex surface; (**b**) PS spacer/colloidal crystal interface; (**c**,**d**) optical microscopy images of the SiO_2_ opal-like colloidal crystal self-assembled onto the PS spacer: (**c**) convex surface; (**d**) PS spacer/colloidal crystal interface; (**e**,**f**) optical microscopy images of the SiO_2_ opal-like colloidal crystal self-assembled onto the PS spacer, sunk into water: (**e**) convex surface; (**f**) PS spacer/colloidal crystal interface; (**g**,**h**) UV-Vis reflection bands’ variation spectra with sucrose concentration: (**g**) convex surface; (**h**) PS spacer/colloidal crystal interface.

It seems that a hydrophobic substrate favors the ordering of sub-micron spheres in its vicinity. The SEM investigations show a better organization of the external surface (Figure 5a) and a much better organization for the interface (Figure 5b). The optical microscopy investigations show a minor change of the external surface (Figure 5c) and a light bluish color with some dispersed small green spots of the interface (Figure 5d). The quality of the self-assembled colloidal crystal became evident when both surfaces changed their color, when the crystal was sunk into water (Figure 5e,f). The UV-Vis measurements performed on the crystal surfaces when it was sunk in a water/sucrose solution showed the expected red shift of the (111) reflection band (Figure 5g,h). However, the crystal sensitivity to refractive index variation (Δλ/Δn) of the sucrose solution was rather weak. We measured 115 nm/RIU (nanometers/refractive index units) for the convex surface and 90 nm/RIU for the concave surface, values which are comparable with those measured by other researchers on colloidal crystals self-assembled on optical fiber tips, 108 nm/RIU [13], but which are much lower than those obtained with set-ups in which the surface plasmon resonance effect was used, 1877 nm/RIU [11]. Moreover, if the measurements are performed through the optical fiber, the colloidal crystals detach rapidly (in a few seconds) from the fiber tip when they are sunk into liquids and, thus, in this form, the silica convex colloidal crystal cannot be used as a self-assembled sensor on an optical fiber tip.

**Figure 6:**
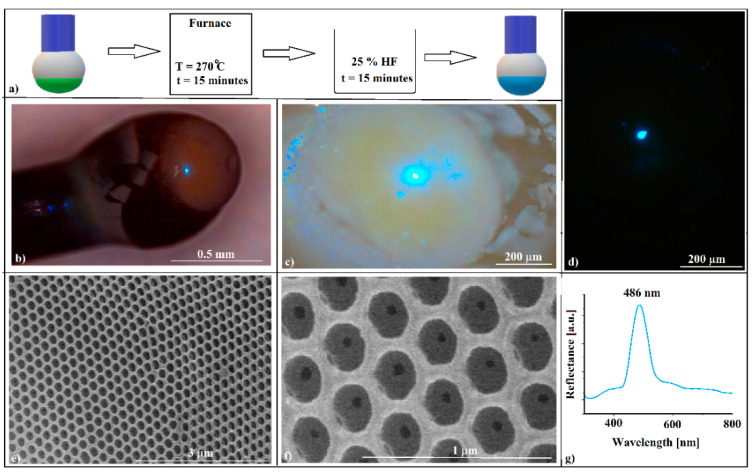
(**a**) Schematic of a polystyrene inverse-opal crystal fabrication on the optical fiber tip; (**b**–**d**) optical images of polystyrene inverse opal; (**e**,**f**) SEM images of polystyrene inverse opal (convex surface); (**g**) UV-Vis retro-reflection spectra (from outside) of the inverse-opal crystal self-assembled on the polystyrene spacer fabricated on the optical fiber tip.

However, the adherence of the photonic crystal to the optical fiber can be solved by transforming the opal into an inverse opal crystal. The heat treatment of the silica opals self-assembled onto polystyrene spacers in a furnace at T = 270 °C for 15 min, followed by immersion in a hydrofluoric acid solution for 15 min, washing in distilled water, and natural drying (Figure 6a), leads to the formation of an inverse-opal-type structure attached to the optical fiber head. A problem that might occur at this stage is the loss of the physical integrity and optical properties of the optical fiber. Not every type of optical fiber is capable of withstanding 270 °C. Furthermore, we must point out that convex silica opal, polystyrene spacer, and polystyrene inverse opal can be obtained through the hanging drop method by using any other kind of fiber, such as a simple metal wire.

After the heat and chemical treatments, the structure seems to retain its volume and shape (Figure 6b). The color change from green (Figure 2a,b) to bluish (Figure 6b–d) is an indication of the conversion of opal to inverse opal and that a good-quality inverse opal can be obtained in this way. The SEM images in Figure 6e,f confirm this assertion. The acquired UV-Vis spectrum (Figure 6g) from the outside surface of the crystal shows that the colloidal crystal changes its maximum reflection band to lower wavelengths (from 555 nm to 486 nm), a phenomenon that is specific to the transition from opals to inverse opals, the 486 nm corresponding quite well to the (111) family planes of a polystyrene/air inverse opal with air holes of 264 nm. By using 1.59 for polystyrene (instead n_SiO2_) and 1.00 for air as refractive indices values, as well as f = 0.26 as the filling factor, we obtained, according to Equations (2) and (3), a theoretical bandgap, λ = 497 nm, which is close to our measured value.

**Figure 7:**
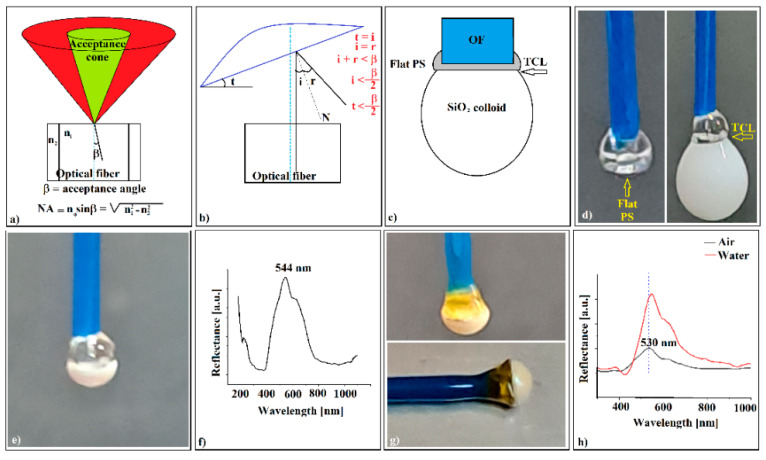
(**a**) Schematic of the optical fiber acceptance angle; (**b**) schematic of optical fiber/colloidal crystal tilt and acceptance angles;(**c**) schematic of optical fiber/PS spacer/colloid drop; (**d**) images of a flat PS spacer and triple contact-line zone of a colloid drop deposited onto the PS spacer; (**e**) image of axisymmetric opal-like colloidal crystal self-assembled on PS spacer on the optical fiber tip; (**f**) UV-Vis spectrum (through the optical fiber) of the SiO_2_ opal-like colloidal crystal; (**g**) images of PS-infiltrated SiO_2_ opal (vertical fiber) after sample thermal treatment, and PS inverse opal (horizontal fiber) after HF treatment; (**h**) UV Vis retro-reflection spectra (through the optical fiber) of the inverse-opal axisymmetric self-assembled on a flat PS spacer on the optical fiber tip.

Unfortunately, the convex colloidal crystal self-assembled on the optical fiber tip could not be used, either as opal or as inverse opal, as an opto-chemical sensor. Even though the adherence to the optical fiber was solved (the water-immersed device resisted ultrasonication and could not be detached without destroying it), the UV-Vis retro-reflection (through the optical fiber) was absent. The reason for this was the lack of alignment between the crystal and the optical fiber axis. As shown in Figure 7a, the light that can enter an optical fiber is limited to an acceptance cone defined by the acceptance angle β [29]. If a white light beam emitted through an optical fiber encounters a photonic crystal capable of Bragg diffraction (i = r) tilted at an angle, t (Figure 7b), it is diffracted and reflected at angles related to the wavelength. In the optical fiber, however, only beams falling inside the acceptance cone are captured. If the crystal is inclined at an angle greater than half the acceptance angle, no reflection band specific to the photonic crystal can be recorded by retroreflection by the spectrophotometer. Moreover, if the optical fiber tip is submerged in a liquid, its refractive index proportionally reduces the acceptance angle.

This is a problem because the acceptance angle β is quite small for most optical fibers. The optical fiber used by us has a numerical aperture, NA = 0.39, which corresponds to an acceptance angle of β = 23° and, implicitly, a maximum crystal-tilt angle of about t = 11.5°, which is relatively small. During the experiments, we found that the colloid droplet deposited on the polystyrene spacer at the tip of the optical fiber self-assembles into an axisymmetric colloidal crystal with fiber (t < 11.5°) if and only if a droplet with maximum colloid volume is deposited on a flat surface of the spacer (Figure 7c). We suppose that this result was due to the different position of the TCL (at the edge of the flat surface of the spacer, Figure 7c,d) compared to the set-up from Figure 1g, in which the opal was grown from the colloidal drop directly hanging on fiber. In the case in Figure 1g, due to the hydrophilicity of the glass OF in direct contact with the colloidal solution, the TCL fluctuates vertically, whereas, in the case of the introduction of the flat PS hydrophobic spacer, the TCL is strictly constrained at the air–polystyrene–water border, with the only option to horizontally fluctuate. Proceeding in this way, forming a polystyrene spacer, which had a flat bottom face (Figure 7d—left), on the tip of the optical fiber, and on which we deposited a colloidal SiO_2_ drop (Figure 7d—right), we obtained, after the evaporation of water, a colloidal photonic crystal axisymmetric with optical fiber (Figure 7e). The UV-Vis spectrophotometry, which was performed, this time through the optical fiber, showed the existence of a reflection band at λ = 544 nm (Figure 7f), placed in a position close to that generated by the external surface of the crystal, at λ = 555 nm (Figure 2d), and even closer to that given by the surface formed at the interface with the optical fiber when the colloidal crystal was detached from the optical fiber spacer and investigated independently, at λ = 550 nm (Figure 5h). This was because of the poorer quality of the surface formed at the OF/SiO_2_ opal interface than the external surface. Next, performing the polystyrene-spacer-infiltration operation at T = 270 °C on the colloidal crystal, as shown in Figure 7e, we obtained an “infiltrated opal” (Figure 7g, vertical fiber), about which we can remark its golden color, a structural color theoretically predicted by the Bragg equation to be located at λ = 626 nm, and generated by light diffraction on the (111) plane family of a planar colloidal silica crystal infiltrated with polystyrene. Again, the inferior quality of the interface (lower filling factor, f) shifts the theoretical color from yellow-red to yellow (a few tens of nanometers to the lower wavelengths) in the interface area, and much less on the outer surface. After the dissolution of the silica spheres in HF, the photonic crystal of the inverse opal type acquires a gray color, except for the blue spot, which can be seen anywhere on the surface of the inverse opal, depending on the angles of incidence and the observation of the reflected light (Figure 7g, horizontal fiber). Note that all the images and spectra in Figure 7d–h refer to the same sample, so that the exact changes induced by the performed operations can be followed. The UV-Vis retro-reflection (Figure 7h) measurements through the optical fiber on the colloidal crystal of the inverse-opal type formed on the tip of the optical fiber show a broad band centered at λ = 530 nm, which is quite far from that given by the outer face of the crystal, λ = 486 nm. We consider that this difference could be due to the incomplete dissolution and extraction of SiO_2_ near the polystyrene/fiber optic interface. Under this assumption, Equation (3) is rewritten as a system,

(4) $$n_{eff}=\left(1-f\right)\cdot n_{PS}+X\cdot n_{air}+Y\cdot n_{{SiO}_{2}};\;\;\;X+Y=f=0.74\;\;$$

whose solution gives us a value of *Y* = 21%, as the concentration of SiO_2_ remaining in the inverse opal voids. This is not the percentage of the initial volume (mass) of the silica opal, but only the concentration seen in the volume (about 10 layers) at the interface with the optical fiber. The ultrasonication of the sample in HF (its mechanical resistance allowed this operation) did not lead to a significant decrease in the silica residues at the interface. However, used under these conditions (self-assembled axisymmetrically on the fiber-optic tip) as a sensor of the refractive index variation (measurement performed by retro-reflection through the fiber optic), the polystyrene reverse opal shows an even lower sensitivity than in the case of the SiO_2_ opal detached from the optical fiber, namely a sensitivity value S = 40 nm/RIU. It seems that colloidal crystals self-assembled on the head of an optical fiber as a refractive index variation sensor are not the most suitable devices for this purpose. However, although our results are not encouraging for the field of opto-sensors, they open the possibility of designing experiments based on fibers, self-assembly phenomena, and thick colloidal photonic crystals. So far, almost all the experiments concerning the self-assembly of sub-micron spheres in colloidal solutions have resulted in thin photonic colloidal crystals: single layers, bilayers, or few layers of spheres self-assembled in hcp or fcc (face-centered cubic) lattices. The vast majority of these exploit the diffraction effect of light by transmission or reflection and, in particular, Bragg diffraction (the angle of incidence is equal to the angle of reflection). The main reason for this could be that in this way, a wide range of cheap and easy-to-manufacture optical sensors can be synthesized, in which the response given by the change in the effective refractive index (*n_eff_*) in Equation (2) would be as fast as possible, implying the use of thin colloidal films in which the fluid’s infiltration process would also be as fast as possible. From an optical point of view, about twenty layers seem to be sufficient to have a good reflectance on the faces of the crystal or a sufficiently good transmittance. All the additional layers seem to make no contribution to the final result. However, a thick colloidal crystal (hundreds and thousands of layers) has much better mechanical resistance than thin films and can, thus, be used as a free-standing device. The disadvantage of being extremely difficult to impregnate by fluids can turn into the advantage of being able to be used in experimental set-ups in which they can be moved inside a fluid (opto-fluidics), because they can achieve this without disintegrating. Additionally, unlike a thin film, a thick colloidal crystal can be “shaped in the third dimension,” i.e., convex, concave [30], or tilt surfaces (prisms [31]), where the wave optics meet the geometric optics (focus, non-Bragg diffraction) strongly increasing the number of possible applications. The new major topics that have recently appeared, such as small-diameter bent waveguides [32], curved-space nanophotonics [33], or topological photonics [34], require the fabrication of 3D nonplanar photonic crystals, which, by means of proper experimental set-ups can be used to verify the issued theoretical hypotheses.

## 4. Conclusions

High-quality convex colloidal photonic crystals can be grown on the tip of an optical fiber by self-assembly from a silica sphere (264 nm) colloidal solution using the hanging drop method.

These crystals are convex-shaped, produce the diffraction of reflecting light with high efficiency (blazing colors can be seen on optical microscopy), and have a high curvature (they are ~800 μm in diameter and tens of micrometers in thickness). These characteristics could make them useful as convex diffraction devices in imaging spectrometers.

The outside UV-Vis retro-reflection spectra of these crystals show a reflection maximum placed at the wavelength, λ ~555 nm, corresponding to the Bragg diffraction of the (111) flat crystal plane family. The optical fiber/colloidal crystal interface is disordered; thus, no light diffraction can be registered.

The ordering at the optical fiber/colloidal crystal interface can be increased by forming a polystyrene spacer on the optical fiber tip, which can also serve as a self-assembly substrate for silica colloid, as a mechanical bond between the fiber and the crystal, and as a filler reservoir for an inverse-opal synthesis.

The silica opal-like grown on the optical fiber tip can be transformed into a high-quality polystyrene (blazing colors) inverse opal by using the polystyrene spacer as the filler.

The colloidal crystal self-assembles axisymmetrically onto the optical fiber tip only if the maximum volume of the colloid drop is settled on a flat end of the polystyrene spacer.

## Data Availability

Data are contained within the article.
